# Toward humanistic healthcare through dystopian visions: Sally Wiener Grotta’s “One Widow’s Healing”

**DOI:** 10.1186/s13010-025-00163-5

**Published:** 2025-02-05

**Authors:** Meeyoung Kang

**Affiliations:** Sookmyung Women’s University, Seoul, Republic of Korea

**Keywords:** Critical medical humanities, “One Widow’s Healing”, Human alienation, Well-being, Humanistic medicine

## Abstract

**Background:**

Critical medical humanities critique the traditional medical humanities’ focus on producing humane doctors, arguing that it plays only a supplementary role in medical education, and advocate for understanding health, disease, and humanity from a biocultural perspective. Essentially, they emphasize structural inequalities in modern medicine.

**Methods:**

This study analyzes Sally Wiener Grotta’s “One Widow’s Healing” from the perspective of critical medical humanities. In line with this critical perspective, this study highlights the human alienation and oppression caused by biopower and technology-driven medicine in “One Widow’s Healing.”

**Results:**

This story presents a dystopian vision of future healthcare systems in the highly technologically advanced and hyper-connected societies of 2100 and advocates for a reorientation of medicine toward a holistic, culturally informed practice that prioritizes human well-being and empathy.

**Conclusions:**

By analyzing the literary response to the dystopian future, this study explores the potential dangers at the intersection of capitalism and technocentric healthcare, reflecting on the future direction of humanistic medicine.

## Introduction

This study analyzes the story of Sally Wiener Grotta’s “One Widow’s Healing” to explore the potential dangers that could arise at the intersection of deep-rooted capitalism and technocentric healthcare. Further, it discusses aspects of which humanistic medicine must be vigilant and where it should aim to progress. Consequently, this study seeks to demonstrate how the medical humanities can transcend the merely supplementary role in medical education and become a means of critically and reflectively engaging with modern medicine and techno-centrism. In this process, medicine that lacks an understanding of ontological human vulnerability and interdependence can have fatal consequences for humans. As a literary resistance and alternative to the dystopian future, “One Widow’s Healing” proposes a holistic concept of healthcare that involves a shift from treatment or cure to healing, from health to well-being, and from passive object to active subject, offering a vision of the human and an ethics of care.

Sally Wiener Grotta’s “One Widow’s Healing,” a Health Odyssey Award winner, is a science fiction story that represents a dystopian vision of future healthcare systems. Set against the backdrop of the year 2100, “One Widow’s Healing” images a future society where technologies such as the Metaverse, AI (Artificial Intelligence), AR (Augmented Reality), VR (Virtual Reality), and IoT (Internet of Things) have become ubiquitous. Maria is a doctor who makes substantial contributions to inventing an electronic medical program in which diagnoses and surgeries can be conducted through medical technology and equipment, without the need for direct meetings between doctors and patients. She has accumulated a large amount of patient data as a nanophysician, collaborating with other doctors of Medbiotics to develop and popularize remote diagnosis and treatment programs. On the surface, the story seems to present a blueprint for a technophilic medical society where the accumulated patient data collected by Maria corresponds with the technological prowess of businessmen and scientists, enables doctors and patients to meet online for diagnosis, and facilitates remote treatment using nanodevices called nanites. The patient care system built by Maria is being transformed into a highly efficient, profit-driven healthcare system by WLS, a monopolistic corporation. In this transition, human existence, reduced to data and statistics, becomes a target for exploitation and surveillance. In a technology-centered society that denies individual autonomy and control, healthcare accelerates discrimination and marginalization against minority groups. As a highly developed technological society unveils its side effects, individuals find themselves not only alienated from technology, capital, the self, and others but also dominated and oppressed by biopower. Such an environment threatens human health and well-being. Maria grapples with these challenges as she seeks humanitarian medicine that protects the health rights of minorities from technology, capital, and power.

Classifying this narrative as dystopian literature allows for an understanding of this work in the lineage of extreme depictions of humanity’s catastrophe found in texts such as Aldous Huxley’s *Brave New World* (1932) and George Orwell’s *1984* (1949). These novels shockingly illustrate how states and power institutions exploit advanced technology to suppress human freedom and individuality. Published in different eras, these works predict a future that culminates in totalitarian societies, warning that advanced technology will become tools of oppression. In “Dystopian Literature and the Sociological Imagination,” Sean Seeger and Daniel Davison-Vecchione analyze the concept of dystopian literature as a “thought experiment” within social theory. They emphasize that dystopian novels frequently provide grounded narratives that mirror social and historical forces, which shape the personal experiences of characters within oppressive regimes. This interplay allows readers to explore the effects of real-world social, technological, and political trends on individual lives, making dystopian works powerful tools for examining possible societal consequences of current practices [1]. Likewise, “One Widow’s Healing” deeply explores the health issues of modern society through its dystopian elements. This work does not conclude with Maria’s complete defeat or unilateral victory in her struggle for human-centered care in a society dominated by surveillance, exploitation, marginalization, and discrimination. Instead, it ends with her escaping her long-isolated apartment, defying WLS’s opposition. This open ending, reflecting on the dystopian future, encourages readers to reconsider contemporary society and healthcare, ultimately placing the responsibility of reshaping the future in their hands.

The study’s narrative goes beyond simply using medical issues as a literary theme or subject; it embodies the intrinsic goals of medicine and literature, both of which reflect the contemplation of human life and reality as well as emotional empathy for the deep scars and traumas left on the human soul and body. The statement “Medicine is the most humane of the sciences, the most scientific of the humanities” underscores the unique position of medicine at the crossroads of science and the humanities. Modern medicine’s emphasis on a scientific approach to disease and knowledge has faced considerable criticism for its techno-centrism; it “must come to terms intellectually and politically with the human–machine interface to have confidence about the aspects of care than only a human can provide and to manage rather than be managed in the human–machine relationship.”^6^ Illuminating the dynamics of the relationship, “One Widow’s Healing” advocates the reorientation of medicine toward a holistic, culturally informed practice that prioritizes human well-being and empathy.

Ever since the first literature major was appointed professor at the University of Pennsylvania Medical School in 1972, literary scholars in North America have been at the forefront of the medical humanities movement. Initially, the medical humanities emphasized the importance of humanistic education and literacy, which could assist medical practitioners in their practice. However, over time, the idea that the relationship between doctors and patients should be human-centered has been increasingly emphasized. Subsequently, various literary works have been produced to fulfill educational requirements and cultivate the empathy necessary for medical practitioners to treat patients. However, recent critical medical humanities movements have recognized tensions within the field of medical humanities and extended beyond the humanistic doctoring thesis, which has been the central tenet of traditional medical humanities. Such movements have sought to expand the humanistic agendas of health and healthcare systems. Consequently, critical medical humanities deal with the global dissemination and influence of medical-related scientific technologies, the operation of power ideologies related to health and medicine, and various inequalities and injustices in the medical field.

Initially, critical medical humanities posited that by focusing on the pragmatic goal of training humane doctors, medical humanities merely played a supplementary or instrumental role in medical education. Therefore, the “critical” part of critical medical humanities signifies the need for more intensive engagement in the medical humanities: “how health, illness and treatment are constituted in and through tangled webs of human and non-human biosocial organisms, political-economic formations, discourses and affects” [2]. Transcending the dichotomy between biological, sociocultural, and critical medical humanities suggests that health, disease, and humanity itself should be understood from a biocultural perspective. This emphasizes the structural inequalities in modern medicine that alienate certain subjects and produce only specific types of knowledge. By aligning with fields such as science and technology studies, disability studies, queer theory, cultural studies, and media studies, critical medical humanities can re-conceptualize health, disease, and medicine within multicultural and global contexts and networks critically and creatively. From the perspective of critical medical humanities, this study aims to analyze the work “One Widow’s Healing,” discussing the dynamic interactions and inherent tensions among technology, capital, and the effects of the surrounding healthcare.

The critical, macro perspective on the structural, social, and political elements of healthcare in “One Widow’s Healing” merges with the micro perspective of narrative medicine. This embodies a humanistic approach to medicine that relies on close reading and empathy within the patient–physician relationship, rooted in the principles of narrative medicine. Rita Charon champions narrative medicine in *Principles and Practice of Narrative Medicine* (2017) as a means of fostering deeper understanding between patient and physician through personal stories and experiences. She underscores that narrative competence—the ability to listen to and interpret patient narratives—enables physicians to appreciate the unique contexts of patients’ lives and empathize with them [3]. Likewise, “One Widow’s Healing” portrays how empathy through personal narratives between Maria and her patient, Jo, transforms them both, highlighting the essential human experience of patients in the healing process. This vision diverges sharply from a clinical environment dominated by computer-generated questions, actions, and responses, or by a healthcare system seeking complete control over the clinical encounter.

## Human alienation in a hyperconnected world

This story is set in a future where all aspects of human life operate through AI, AR, VR, and IoT; everything humans need is delivered by drones, and all medical services are provided remotely online using nano-sized small devices called nanites implanted in people’s bodies. Set in what resembles a techno-utopia, “One Widow’s Healing” represents many changes in how humans act and think, portraying a society in which convenience and efficiency are maximized through science and technology. However, the expectations of such convenience and efficiency transform into dystopian imagination as the story unfolds, illustrating that not only Maria, the doctor who contributed to developing the future remote healthcare system but also her patients—those who benefit from this technology—experience surveillance and alienation owing to the impersonal nature of remote technology and empathy-deficient medicine. This depiction serves as a reminder of the humanistic values that current and future medicine should not overlook. The narrative offers a critical perspective on the reversal of reality and virtuality, the inversion of technology and humanity, and technological authoritarianism in a society in which people rely too heavily on images and data than on humans—which is regarded as secondary.

The most striking aspect of society in “One Widow’s Healing” is human alienation. Extreme efficiency and convenience eliminate the need to interact with others. In the story, Maria did not leave her house for 13 years after her husband’s death as “all information, entertainment, and interactions were online. Anything she needed or wanted could be 3D printed or delivered by drones” [4]. Like everyone else, she became too content within the confines of her safe, well-provisioned apartments. She even decided to forego travel plans and attend important online events in this hyperconnected society. Thus, in such a society, humans become increasingly alienated from others and themselves to the extent that an avatar replaces them in every aspect of life.

The problem is that while the online world replaces the real world, the virtual existence of avatars not only replaces human beings but also attains an existence and authority that surpasses human existence:

Dr. Maria Heilari fidgeted with her avatar’s clothing, editing it up to the last minute despite the rental agreement that forbade tampering with the design. Regardless of what Gabrielle, Chanel’s virtual saleswoman, had said, the outfit was too frivolous, even ridiculous, just when Maria needed the world to take her seriously. ... Why did I let Gabrielle talk me out of renting a traditional blazer, turtleneck and pleated pants like my old standby business meeting outfit that must be somewhere in the back of my closet? (p. 1)

Rather than replicating her being, Maria’s avatar possesses authority and value that transcend her existence. Although Maria finds her avatar’s presence crude when the world should take her seriously, she cannot modify its design owing to the contract that prevents her from altering it. The avatar shares Maria’s legs, face, and hairstyle, but its existence experiences a reversal because it holds its independent meaning that cannot be altered. This shows that humans lose agency and autonomy when they are alienated from their own corporeality and materiality.

Moreover, human values are alienated by the power of capital, which commodifies human existence. Embodying artificial, mechanical, and inhuman values in opposition to Maria’s worldview, the founder and CEO of Medbiotics, Dr. Lamont Mitchell, and his partner Dr. Kamau Quammen use “Maria’s meticulous analyses of over twenty million patient records collected over her decades-long career as a doctor” (p. 13) to create a new, commercially viable medical program and platform. The doctors’ claim that they invented Cyberhands—a product of the Whole Life System (WLS) company—to practice a form of humanistic care based on human individuality and values, but they utilize Maria’s research only for military purposes. Although Mitch and Quammen claim that the program aimed to “fix the broken health system for everyone” (p. 13), Maria dismisses it as “bullshit” (p. 8) as this innovation presupposes that every individual should be connected to the system in a hyper-connected society that ultimately reinforces human alienation. In such a society, people are digitized and transformed to directly connect to computer networks.

The problem with this process is that it objectifies humans as tools for producing and homogenizing data and alienates individuals’ emotions and values. Through the processes of objectification, “what medicine ignores is the patient’s subjectivity” [5]. The data Maria has collected over the years include not only vast amounts of intimate biometric information and personal identity details but also information capturing the complexities of human emotion, cultural nuances, and subjective experiences. Despite the impossibility of converting human emotions and values into data, human datafication reduces the human to a mere object that generates data, leading to commodification and reification of human values.

In this digitized society, “One Widow’s Healing” illustrates the shift in human existence and the medical environment, where humans are datafied through information collected via IoT and wearable devices and reduced to unique identification numbers, and where the biological basis of human existence is increasingly negated. As Henk Ten asserted, human beings will be digitized, and everybody’s genetic constitution will be digitally accessible to enable precise and individualized care offered by robots [6]. The transformation into data to benefit from efficient medical services results in becoming a victim of inhumane care, which is evident in technology-centric, efficiency-driven WLS. Efficiency is the ratio of the output to the inputs of any system. An efficient system or person achieves higher levels of performance relative to the inputs (resources, time, money) consumed [7]. In this system, WLS requires doctors to treat patients strictly according to the manual, leaving no room for personal or humane interactions. Nonetheless, Maria remembers her patients by their names and faces in a healthcare system where each patient is processed by a unique identification number, believing “they were her life” (p. 7), and tries to consider each patient’s feelings and circumstances with care. However, she is chastised by WLS for “unprofessional conduct” (p. 7) as she is not allowed to engage in social diversions when checking diagnostics and providing prescriptions.

In the medical system of “One Widow’s Healing,” the relationship between people operates on efficiency rather than human values. As human values become fully data-driven and the processes of treating and managing individuals are digitized, the patient–doctor relationship is increasingly evolving into a mechanical one that prioritizes high efficiency. In a society where the human body is reduced to images and human values are transformed into data, Maria’s humanistic approach to medicine ultimately fails to make a lasting impact against the bio-power of future society. However, in the dystopian vision of future healthcare in which humans become alienated from their sense of self, others, their own physicality, and human values, losing their authority and power as independent agents offer a moment for reflection on today’s medical practices and raises concerns about the direction of future healthcare systems.

## Economic/biopolitical repression in capitalist healthcare

“One Widow’s Healing” represents economic and biopolitical repression and control as the negative consequences of a new form of medical service in a hyperconnected society. In such a society, where datafied individuals’ bodies and medical information are transmitted and managed from devices to some location, the expansion of telemedicine not only leads to the easy exposure of individuals’ most intimate information and data, but also implies the potential for evaluating human worth, monitoring daily life, and suppressing freedom of movement. In particular, the medical technology and systems of multinational corporations that have rapidly expanded owing to the influence of capitalism and neoliberalism create the foundation for digital panopticons, where biopolitics operates extensively. Similarly, in “One Widow’s Healing,” technology is used by those in power to exert omnipresent control over the population, leading to economic and biopolitical repression.

The future society depicted in “One Widow’s Healing” is an intensified reflection of the characteristics of late capitalism as described by Frederic Jameson, that is, the proliferation of new forms of transnational organizations and the affiliation of government and big business [8]. This late capitalist society is characterized by an unprecedented level of mass production, resulting in ever-greater profit margins for multinational corporations and a frantic economic urgency to produce fresh waves of novel goods for the capitalist takeover of human lives. Likewise, the society in “One Widow’s Healing” is predominantly driven by extreme capitalist values. The notion of capital, by which one must extract maximum utility from something, generate maximum profit from investments, and more effectively control objects and people with minimal costs, has penetrated human lives on a micro level. According to David Harvey, the emphasis on privatization and market-driven approaches has led to commodifying healthcare services. This has resulted in prioritizing profit over patient care and limiting access to healthcare for marginalized populations while enriching private entities [9].

Maria’s humanist research on her patients’ data is utilized to maximize profits by Medbotics, the multinational corporation of medical devices and WLS with a global platform; this creates a situation where every person’s participation is inevitable. These corporations prioritize profits over corporate social responsibility and play the role of “shadow sovereigns” [10], comprehensively influencing citizens’ lives. The dominance of multinational corporations over ordinary citizens implies that governments’ roles and powers are replaced by capitalist powers. Particularly, shadow sovereigns in the field of medicine, which should be managed as a public area where human health and wellness take priority over private interests or profits, threaten human rights.

In the story, Medbiotics changes people’s thoughts and behaviors through innovative medical technology and market dominance. Operating globally, its headquarters is in the same 100-floor Jinghai Tower that houses WLS’s Asian offices and Mitch’s penthouse, highlighting the close relationship between transnational business and government systems. This close relationship is also illustrated by the fact that the CEO of Medbiotics (Mitch), received an award from the president of the United Medical Association for the substantial contributions the company has made to the field of medicine. After expanding its influence through various medical devices, Medbiotics celebrates its success in automating and digitalizing medical systems by utilizing Maria’s data research and leveraging the global platform of WLS. Mitch and his partner, Kamau Quammen, take pride in revolutionizing the medical field through various cybermedical inventions, making their founding investors wealthier. After introducing their products such as the Organ Replacement 3D Printer, Remote Holographic Surgery, Robotic Nurses in hospitals, and Lifelong Diagnostic and Treatment Nanites, they boast about Medbiotics’ Cyberhands as “one of the most important medical breakthroughs of this century.”

However, Cyberhands is a program that distorted Maria’s research—which emphasized the importance of sensory interaction between people—from having a humanistic perspective to having a capitalist perspective. It focuses on treating more patients cost-effectively in a short period. As a result, pursuing universal human values leads to classifying humans based on economic value, so that treatment methods and life expectancy are determined accordingly. This is illustrated when Maria attempts to find the appropriate treatment for Joe, a low-income patient diagnosed with small-vessel cerebral aneurysms and performs remote surgery and prescriptions.

She tried to add Nanotros to Joe’s daily meds to prevent future aneurysms, but an UNAUTHORIZED warning box strobed on her screen. Nanotros was a high-cost anti-modulator considered unsuitable for the 28% of patients who couldn’t afford supplemental plans-the so-called Lifers- like Joe. Damn bureaucrats! (p. 9)

This society, where low-income patients are classified as “Lifers,” is a place where biopolitics has become entrenched to the extent that patients are restricted from receiving appropriate medical treatment. The term “Lifer,” originally referring to prisoners serving life sentences, implies low-income patients’ subjection and subjugation. Similarly, the WLS symbolizes the inescapable biopower that persists throughout life, demonstrating that society is manipulated by Foucauldian biopolitics.

According to Foucault, the beginning of modernity marked the emergence of biopolitics, as opposed to sovereign power, which began limiting monarchs’ actions. Biopolitics succeeded in aligning the “phenomena of population” with economic processes and integrating human bodies into the “machinery of production” [11]. This gave rise to “a policy of coercions that act upon the body” and “a political anatomy” that defined how one may exert control over others’ bodies [12]. This process has led to governance methods that treat individual human bodies as mechanical devices, with the goal of achieving economic optimization and political compliance. Political anatomy, based on human life and welfare, establishes rules and governance mechanisms by collectively engaging disciplines and populations at mathematical and statistical levels. Consequently, human collectives, captured through birth rates, mortality rates, life expectancies, and other quantifiable measures, are reduced to numbers and statistics as governance targets. This defines “what must live and what must die” [13], showing how all individual lives are subjected to being ruled through biopolitical control.

“One Widow’s Healing” literarily represents the process by which victims of Foucault’s biopolitics within the medical environment are conceptualized as vulnerable bodies. The fact that the program does not provide potential life-saving treatment to those with low economic productivity demonstrates that the healthcare system in this society operates in a way that kills—rather than saves—people. Here, “One Widow’s Healing” captures a critical shift in the understanding of vulnerable bodies in healthcare not only as bodies made vulnerable by disease or trauma but also as those rendered vulnerable by social forces and structures. This resonates with Judith Butler’s definition of vulnerability as “the greater likelihood of dying for those who are marginalized, understood as the fatal consequence of a pervasive social inequality.” Vulnerability is regarded “as part of social relations, even as a feature of social relations” [14]. Likewise, Gámez-Fernández and Fernández-Santiago (2024) asserted that “[n]obody is invulnerAble” [15] as social forces, such as government intervention and the distribution of medical resources, shape various forms of vulnerability. The dystopian society in “One Widow’s Healing” shows that “vulnerability is shared, that it is common property, and that it allows for a vision of the human as essentially interdependent and in no way autonomous” [16]. Ultimately, the realization that both patients and doctors are vulnerable and subjugated in this society proposes a new form of medicine governed by the ethics of care.

Indeed, enraged at the biopolitical repression producing vulnerable bodies with a greater likelihood of dying, Maria sets up a prescription for “auto-repeat treatments as needed” (p. 9). However, at that moment, “a large, thick-necked man in a Medbiotics logo shirt scowled at her” and insisted that she “terminate this vidcon immediately” (p. 9). As her connection to Joe closes before she can say goodbye to him, Maria becomes strongly suspicious that the man has just taken over her computer. Then, Maria decided to express her opposition to Cyberhands at the product launch stage, which would be watched by everyone. However, in this hyperconnected society, where every move is monitored, her decision has already been leaked. As she tries to voice her opinion, identity theft occurs, causing Maria to lose her agency and autonomy:

Damn, their cherry-picking twisting of my discovery. But no more. Tonight, I’ll finally tell the world: WLS’s accursed cyberhands aren’t the answer. ... Maria took a deep breath, then started to respond, “Touch is perhaps the most important of our five senses because it’s more than...” But the words that came out of her avatar’s mouth weren’t Maria’s. ... The avatar had been hijacked! Maria poked icons and buttons, frantically trying to regain control, to no avail. Not knowing what to do, Maria kept hitting the same commands. Suddenly it was over, and the next question was directed to Mitch. Maria crumbled in defeat, slouching deep into her high-backed desk chair, tears of frustration and anger pouring unchecked. (pp. 14–15)

While Maria does not know who is watching what, she feels she is being monitored and realizes that her suspicions are true. One day, a man named Alex visits Maria’s apartment, proposing that she work with his boss, Methany. Alex already knows how the WLS has deceived and exploited Maria, and hints that his boss is interested in establishing the humanistic medical care system that Maria aspires to build. He tells Maria that he and Methany tried to contact her about the possibility of working together but were repeatedly blocked from contacting her. Alex says to Maria, “You do realize they’re watching you? They know I’m here, and are probably listening… Some anonymous hacker has blocked me every other time I’ve tried to reach you” (p. 18). Even while meeting with Alex, Maria continues to be interrupted by calls and interferences from her headquarters until she is ultimately restrained by security personnel.

This situation illustrates that Maria lives in a Digital Panopticon in which oppressive surveillance and control are at work. Drawing on Jeremy Bentham’s concept of the panoptic architectural figure, Foucault explained his philosophical theories metaphorically. A panopticon is a circular structure with a central guard tower and peripheral cells, allowing the observation of all the cells from the tower [13]. Blinded windows in the tower ensure that inmates never know when they are being watched, and their behavior is controlled through perceived surveillance. This leads inmates to internalize constant surveillance, such that they exhibit “normal behavior” consistently. Such panopticism relates to biopower as surveillance facilitates discipline over the body and life because “the prisoner in the Panopticon and the patient at the end of the stethoscope both remain silent as the techniques of surveillance sweep over them” [17]. A hyperconnected society serves as a digital panopticon in which personal data collected are ultimately used to decide and restrict freedom by creating profiles for people using databases. At the intersection of health and biopolitics, “the medical discipline is part of the whole process, and the medical episteme can be seen to exhibit the same kind of processes and power mechanisms” [18].

In “One Widow’s Healing,” WLS, which claims to be a comprehensive online healthcare system, turns out to be a surveillant digital Panopticon from the perspective of a technocentric future hyperconnected society, particularly in the medical field. In this society, surveillance is a daily and natural occurrence, and people are unaware of who watches them, where, or how. As a result, individuals internalize the fact that they are being watched and assume the role of their own guards. Consequently, everything, from when and what people eat to where they go and how they live their routines, is naturally determined; this highlights the interrelationship between panopticism and biopower. The routines people believe are for their safety and health and have naturally internalized also function to subtly induce and determine people’s deaths through the collusion of scientific and capitalist ideologies in terms of people’s objectification and dehumanization.

## Degradation of human health

In “One Widow’s Healing,” despite highly advanced technology that has maximized convenience and efficiency in human life and led to the development of high-level medical technologies, people’s health has deteriorated. Describing this situation as “one of the great mysteries of modern medicine” (p. 13), Dr. Quammen explains the background of the development of the telemedicine platform using Cyberhands: “WLS-AIs combat the potential for illness and incapacity the moment our internal nanites detect a micro-anomaly, often arresting disease or disability before any symptoms manifest. Yet, mortality rates have continued to rise” (p. 13). Notably, the story seeks the answer to the mystery of the decline of human senses and feelings. In a society where the Internet’s virtual world replaces the real world, and biopolitical oppression intensifies, human senses and feelings are either nullified or negated. Human emotions and sensations often become tools for generating capital because “the affective life of individuals is an object-target of and condition for contemporary forms of biopower” [19]; this biopower “captures the essential powers of individual bodies and their capacity to affect and be affected” [20], usurping the creative, generative, and affective force. Additionally, the visual- and consciousness-centered Internet space tends to overlook sensory and emotional acts as the domain of the body, weakening the coexistence of humans and other beings.

If we consider emotions derived from the human body as affect, the suppression of human senses and emotions causes the colonization of affect. Biopower seeks to “normalize, structure, optimize, and subordinate the forces of individuals to enter them into the machine of the economic system, to make them productive members of society who will happily defend it to the death if necessary” [21]. As an ideology about the body, biopower posits universal, normal bodies and affects, producing negative emotions about entities outside this category and encouraging individuals to accept these definitions as essential and vital. This dynamic is evident in “One Widow’s Healing” through machine-based treatment, human stratification, and the commodification of healthcare, as biopower produces and spreads body-related affects. In this way, biopolitical disciplines meticulously control the body’s operations using affect, creating “subjected and docile bodies” [22] (Foucault 1995, p. 138). This control leads individuals to conform to societal norms and expectations, often at the expense of their body’s abilities.

Biopolitics and hyper-connectivity in “One Widow’s Healing” nullify interpersonal relationships and sensual experiences. Singh, Medbiotics’ Chief Communications Officer, dissuades Maria from physically attending WLS’s product launch ceremony, saying, “I can do nothing about the pressing crowds you’re likely to encounter” (p. 6). After hearing that, Maria decides to stay home as “she’d become too content within the confines of her safe, well-provisioned apartment” (p. 5). After her husband Doug’s death, Maria hides “away in their one-bedroom apartment, just going through the motions of getting up and treating her patients remotely, then going back to bed” (p. 3). Even during the online treatment, she is forced to “follow the routine 10-min script: check the internal nanites’ reading, ask the patient standard questions relevant to the symptoms and test results, then sign off on the prescriptions calculated by the WLS-AI” (p. 7). Whenever Maria tries to engage in private conversations about patients’ personal matters, she faces numerous reprimands for “unprofessional conduct” and hears reminders such as “you’re online to check diagnostics and prescribe, not to engage in social diversion or invade individuals’ privacy” (p. 8). Such a mechanical approach to life and medical routines, along with time constraints and patient grading systems, denies room for considering human diversity or individuality, treating all humans as collective objects and replacing human affect with the principles of capital and machine operation. This healthcare system focuses on technology and capital rather than human-centered care, and it operates based on efficiency rather than human values.

Consequently, the decline in humans’ capacity to affect and be affected causes a decrease in bodily functions and immune strength. This leads to a failure to achieve equilibrium between mind, body, and spirit and ultimately increases mortality rates. Human senses and emotions are connected, enabling us to exist as interconnected organisms. Therefore, suppressing senses and emotions affects humans’ physical and mental health and well-being. Moreover, treatment replaced by machines and images is devoid of physical sensations and presupposes the absence of the soul and humanity. The focus of treatment has shifted from humans to diseases; technology has become the subject of treatment rather than humans. As a result, people move farther away from achieving their ultimate well-being and lose their body’s abilities.

## Toward human well-being

“One Widow’s Healing” shows that we should aim for well-being—“the state of being or doing well in life in a happy, healthy, or prosperous condition” [23]—rather than just a state of health without illness. This approach pursues healing as the restoration of physical, emotional, or mental well-being rather than merely the removal of the disease. To this end, the narrative offers the potential of affective resistance as a solution to a dystopian future rife with human alienation, biopolitical domination, and the deterioration of human health. Resisting the biopolitical powers that neutralize sensation and the capitalist forces that promote efficiency, Maria embodies “the surpluses of life” that continually escape the techniques governing and administering life [12] in the form of affective resistance. The reason that Maria’s affective flow can be political in this repressive society lies in the fact that “[a]ffects, as intensive forces, are crucial for the capacity to act in order to undermine the stability of molar identities and their oppressive assemblages” [24].

Importantly, Maria’s affect has a political register because, as a pre-emotion and unconscious experience, affect often creates affective dissonance that arises from its incongruity with socially driven emotion [25], producing new perceptions. Moments of disarray reveal what is naturalized and optimized through biopolitics. Maria’s affect as “a set of embodied practices” and “a form of indirect and non-reflective” [26] thinking can serve as a resistance to biopolitics colonizing affect as it offers moments rooted in subjective embodied emotions.

Gazing out her window at the winter-bare trees and granite hiss of Nay Aug Park six floors below, Maria imagined she could smell evergreens and river spray, mixed with the taste of Dough’s flesh on hers. Her mind overlaid years of memories, of hand-in-hand strolls along the park’s twisting paths, through winter snows, spring blossoms, summer breeze, fallen leaves. On one such autumn walk, a scruffy mutt-Watson-had bounded into their path and their hearts. (p. 3. Emphasis Mine)

These moments where sensory experiences and memories converge enable the creation of empathetic relationships with others, providing a counterbalance to oppressive politics that alienate, oppress, and objectify individuals. Maria’s affective flow resists socially accepted hierarchies, fostering empathy and the value of caring for others.

The affective memories that Maria shared with her husband and dog, which continue to live on and communicate with her through a vidcard, allow her to emphasize with her patients and facilitate interpersonal relationships. Maria thinks that Joe is “a good soul who tried to add a friendly touch to their frequent vidcons” (p. 9) as he managed a smile that made his bloodshot eyes sparkle despite the pain. She also believes “Joe and the others deserve better than base-level nutrition, housing and healthcare” (p. 13). Likewise, despite the prohibition on private conversation, she shares Joe’s interests during consultations with questions such as “who won the MegaRegatta lottery today?” (p. 9). This empathetic relationship is based on the belief that the patients need to heal and not just cure their illnesses. This perspective enables Maria to challenge the current healthcare system.

Maria firmly believes that restoring sensory relationships among people is linked to health, well-being, and mortality. Her belief aligns with several theories regarding the relationship between human sensory contact and human health. To illustrate, Stephen Porges’ polyvagal theory offers a compelling perspective on how the autonomic nervous system in mammals—including humans—is influenced by forms of social interaction. According to this theory, eye contact, facial expressions, physical touch, vocal tone, and spoken communication play a role in connecting individuals at an autonomic level. Such positive or safe interactions between humans, or even between humans and other mammals, can help stabilize the nervous system; this may lead to decreased anxiety, depression, hypertension, and related health benefits that arise from in-person and physical connection [27]. Similarly, in *Touching: The Human Significance of the Skin,* Ashley Montague explores the profound role of touch in human relationships and overall well-being. Montagu emphasizes the importance of physical contact—particularly in nurturing relationships—and its impact on mental/physical health [28].

In line with such theories, Maria proposes “instituting old-fashioned house calls,” which fulfill human need for “human touch” and “a sense of human connection” (p. 19) as a solution to a technology-driven healthcare. When Alex raises concerns about costs and logistics, Maria suggests solving the problems using Lifers rather than professional medical staff; this is because she believes that leveraging the “solid empathy rating” of Lifers who exhibit “the highest mortality rates” can inspire patients to embrace life more fully. Likewise, Joseph Mariadhas asserts that “the dynamic functioning of empathy on human behavior in general and interpersonal relations, in particular, recommends that it has to be inculcated and improved for a healthy and wealthy living” [29]. Maria believes that using home-visiting Lifers to establish personal connections—“flesh to flesh”—will engage patients’ psyches, increase their desire and ability to live longer and more fully, and boost their immune systems, ultimately improving their longevity. This belief is based on the fact that “sustaining and growing in interpersonal relationships has a potential quality of increasing psychological well-being and decreasing physical illness of individuals” [30]. Similarly, Maria’s conviction that empathy can lead to well-being by enhancing immunity and vitality is rooted in the importance of mutual relationships centered around social, psychological, and physical well-being.

Eventually, Maria decides to accept gazillionaire Methany’s offer to create a humanistic medical system. To escape the forces of surveillance and oppression, Maria attempts to flee, taking down the vidcard of her deceased husband Doug and dog Watson, and escapes through the rooftop of her apartment. In the end, Maria runs out holding a vidcard that symbolizes sensory affect, which signifies the restoration of humanity, agency, and physicality in future healthcare. Maria’s affect, primarily expressed through an inner monologue throughout the story, serves as a source of strength to protect herself from the dominant powers of surveillance and control and provides opportunities to empathize with others; this implies that the driving force behind her transformation does not come from her knowledge as a doctor or advanced medical technology but from her resonance with others’ marginalization and deprivation as a widow. Despite the biopolitical power that reificates humans, Maria, who has maintained an affective relationship her husband and dog through the vidcard, says the following as she leaves the apartment: “Let’s go. We’ve got lives to heal” (p. 21). Then, “One Widow’s Healing” shows how to instigate a shift from being an object of governance to a self-aware individual, and how to transform healthcare from being capital- and technology-centered to embodying a more humanistic sense of well-being.

## Conclusion

This study aimed to delve into the narrative of “One Widow’s Healing” to investigate the potential dystopian future that may emerge from the convergence of heightened capitalism and focus on technology-driven healthcare. The study examined the aspects of which humanistic medicine should be wary and identified areas toward which it could advance. The narrative of “One Widow’s Healing” delves into the repercussions of an ultra-connected society on human detachment. In a world dominated by AI, AR, VR, IoT, and other cutting-edge technologies, this tale illustrates a community in which technology maximizes convenience and efficiency. However, this techno-utopia morphs into a dystopia as reliance on data and imagery marginalize human elements. In a future society marked by the intensified capitalism, neoliberalism, and hyperconnectivity, healthcare will become a realm where body-related knowledge and emotions are manufactured, vividly showcasing the decline of human values. This story scrutinizes the commercialization of human existence and the process of datafication. Maria’s medical research was exploited for profit by the founders of Medbiotics, who leveraged it to develop a medical platform that reduced humans to mere data-producing tools. This technology-centered paradigm prioritizes efficiency over human care, leaving little space for genuine human connections.

Moreover, the story portrays biopolitical repression and control as adverse outcomes of novel medical services in an ultra-connected society. The proliferation of telemedicine not only exposes individuals’ most intimate information effortlessly, but it also hints at the potential for assessing human value, monitoring daily life, and curtailing freedom based on such data. In particular, medical technology and the operations of multinational corporations, spurred by capitalism and neoliberalism, lay the groundwork for a digital panopticon where biopolitics reigns supreme. Biopolitics views individual human bodies as mechanical instruments aimed at achieving “economic optimization and political compliance,” determining “who should live and who should die.” In “One Widow’s Healing,” the WLS, purportedly a comprehensive online healthcare system, is unveiled as a surveillant digital Panopticon, particularly in the medical domain, where body-related data are vulnerable to surveillance.

Despite the advancements in medical technologies, people’s health deteriorated, prompting the story’s author to explore the root cause of the diminishing human senses and emotions. As biopolitics and hyperconnectivity nullify interpersonal relationships and sensory experiences, people’s abilities to influence and be influenced decline, resulting in weakened bodily functions and immune systems. As human senses and emotions facilitate humans’ connections with others, allowing them to thrive as interconnected beings, the suppression of these elements adversely affects their physical and mental well-being. By resisting biopolitical forces that numb sensations and capitalist pressures that prioritize efficiency, the narrative suggests affective resistance as a remedy for a dystopian future. Maria’s affect, predominantly conveyed through internal monologues throughout the story, emerges as a source of resilience against the dominant powers of surveillance and control and helps her empathize with others.

## Data Availability

No datasets were generated or analysed during the current study.
